# Data on the NADPH-oxidase activity induced by WKYMVm and galectin-3 in bone marrow derived and exudated neutrophils isolated from four different mouse strains

**DOI:** 10.1016/j.dib.2016.12.010

**Published:** 2016-12-13

**Authors:** Lena Björkman, Huamei Forsman, Karin Önnheim

**Affiliations:** Department of Rheumatology and Inflammation Research, Sahlgrenska Academy, University of Gothenburg, Sweden

**Keywords:** Neutrophils, NADPH-oxidase, Formyl peptide receptor agonist, Galectin-3, Mouse strain

## Abstract

Neutrophils are the key players in inflammatory reactions and the release of superoxide through the NADPH-oxidase upon neutrophil activation contributes to bacterial clearance and surrounding tissue damage. Here we describe data on the mouse neutrophil NADPH-oxidase activation induced by the mouse formyl peptide receptor (Fpr) agonist WKYMVm and galectin-3. Neutrophils isolated from bone marrow, peritoneal exudated, and *in vitro* TNFα primed bone marrow neutrophils from four different laboratory strains (C57BL/6, DBA/1, BALB/c and NMRI) were used. Both Fpr agonist and galectin-3 activated neutrophils to release superoxide. No differences were observed in the amounts of superoxide released from neutrophils derived from four different strains.

**Specifications Table**

**Table:** 

Subject area	*Biology*
More specific subject area	*Mouse neutrophils, Inflammation, reactive oxygen species*
Type of data	*Graph, figure*
How data was acquired	Biolumat LB9505 apparatus (Bertold Co., Wildbad, Germany)
Data format	*Analyzed and processed*
Experimental factors	*Bone marrow neutrophils were isolated using density gradient centrifugation and exudated neutrophils were elicited by uric acid injection*
Experimental features	*Neutrophils were pre-warmed together with isoluminol and HRP before activation*
Data source location	*Gothenburg, Sweden*
Data accessibility	*Data is with this article*

**Value of the data**

• This data provides basic characterization of an Fpr agonist and galectin-3 in activating the NADPH-oxidase activation in mouse neutrophils.
• This data compares the neutrophil activation induced by an Fpr agonist and galectin-3 from neutrophils isolated from different mouse strains.
• This data provides information for the Fpr agonist and galectin-3 in activating resting and *in vivo* as well as *in vitro* primed mouse neutrophils.
• This data could be used for further investigation of the molecular mechanism of Fprs and galectin-3 in immune modulation and their role in mouse inflammatory disease models.

## 1 Data

**Fig. 1:**
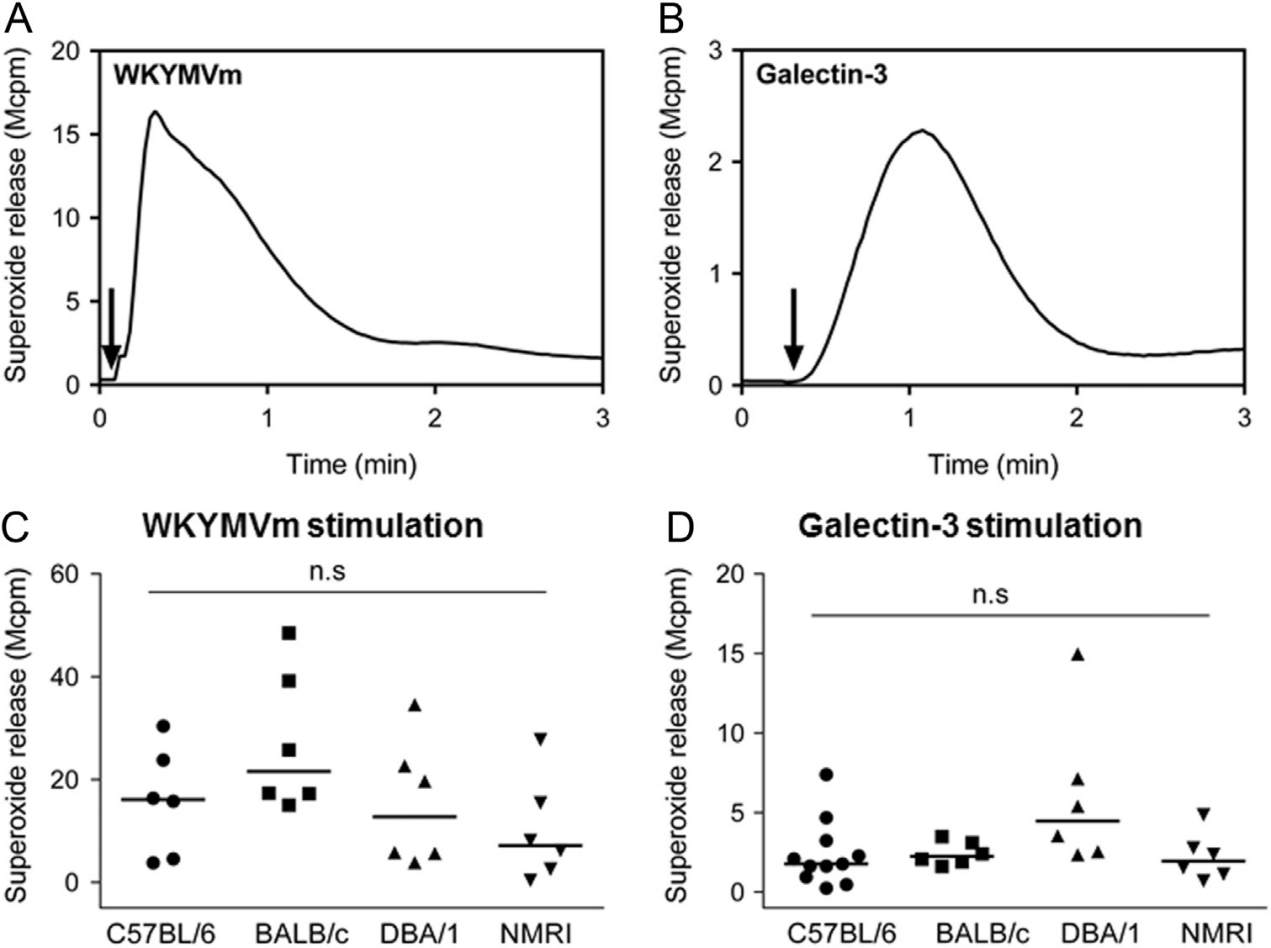
The NADPH-oxidase activation induced by WKYMVm and galectin-3 in bone marrow derived mouse neutrophils. Mouse bone marrow derived neutrophils (5×10^4^ cells/ml) from C57BL/6, DBA/1, BALB/c and NMRI strains were isolated and stimulated with WKYMVm (100 nM) or galectin-3 (40 µg/ml). The release of superoxide was recorded continuously and representative kinetics for WKYMVm (**A**) and galectin-3 (**B**) from the most commonly used laboratory strain C57BL/6 are shown. The arrows indicate the addition of WKYMVm or galectin-3. The data obtained from all four strains are summarized for WKYMVm (**C**) and galectin-3 (**D**) and expressed as the peak superoxide release and median is indicated with horizontal lines. Each symbol represents one individual mouse. One-way ANOVA followed by Turkey׳s multiple comparisons test, n.s, not significant.

**Fig. 2:**
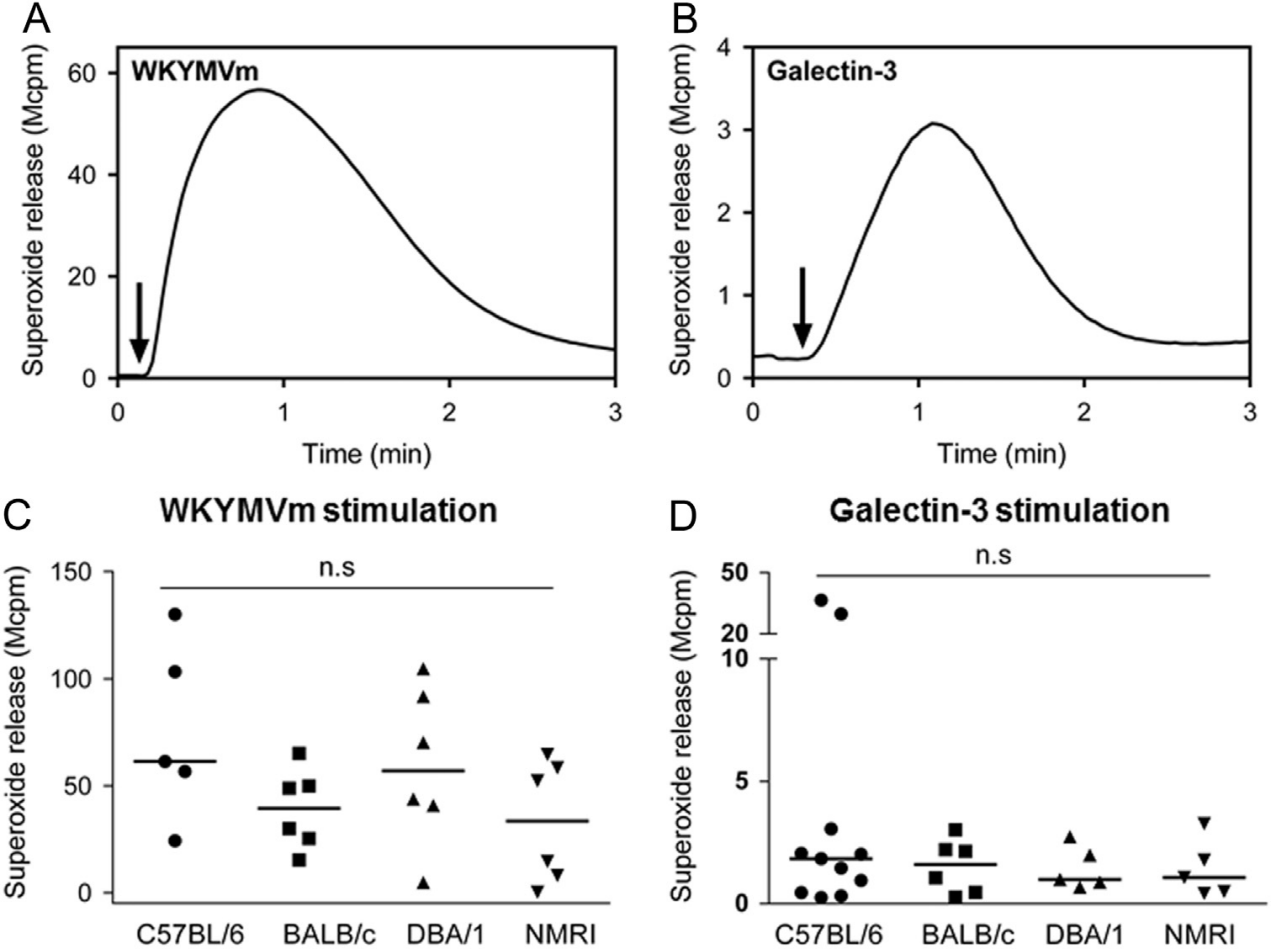
The NADPH-oxidase activation induced by WKYMVm and galectin-3 in peritoneal exudated mouse neutrophils. Peritoneal exudated neutrophils (5×10^4^ cells/ml) from C57BL/6, DBA/1, BALB/c and NMRI strains were stimulated with WKYMVm (100 nM) or galectin-3 (40 µg/ml) and the release of superoxide was recorded continuously. The release of superoxide was recorded continuously and representative kinetics for WKYMVm (**A**) and galectin-3 (**B**) from the most commonly used laboratory strain C57BL/6 are shown. The arrows indicate the addition of WKYMVm or galectin-3. The data obtained from all four strains are summarized for WKYMVm (**C**) and galectin-3 (**D**) and expressed as the peak superoxide release and median is indicated with horizontal lines. Each symbol represents one individual mouse and lines represent medians. One-way ANOVA followed by Turkey׳s multiple comparisons test, n.s, not significant.

**Fig. 3:**
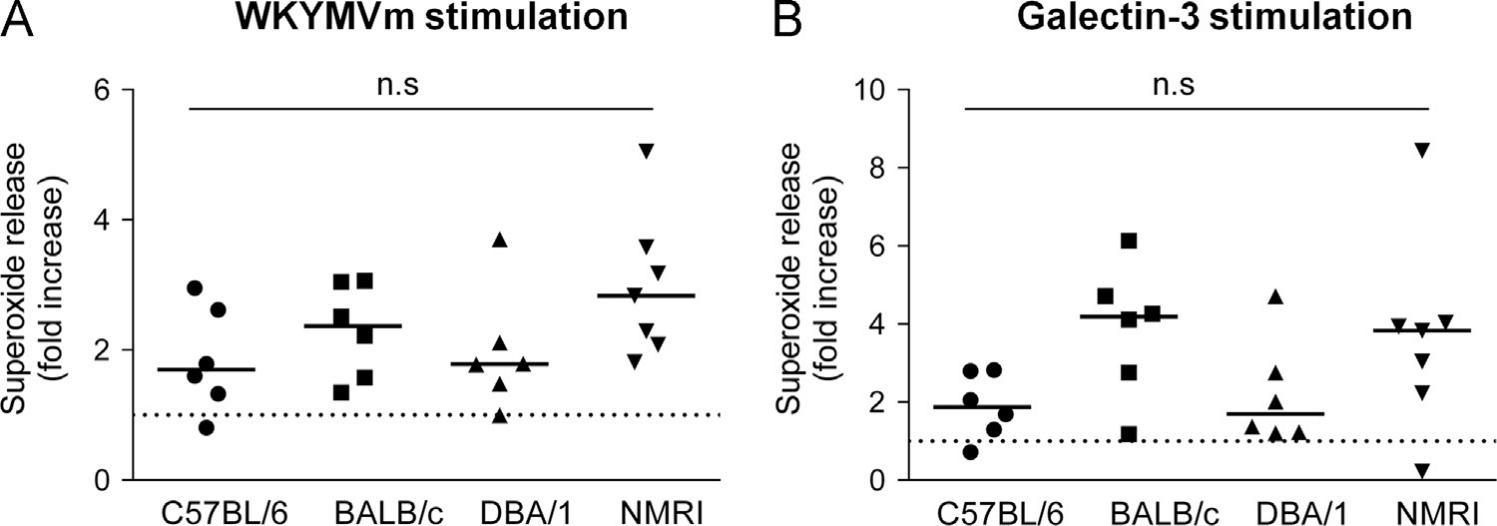
The NADPH-oxidase activation induced by WKYMVm and galectin-3 in TNFα primed neutrophils. Resting bone marrow derived neutrophils from C57BL/6, DBA/1, BALB/c and NMRI strains were in vitro primed with or without TNFα (50 ng/ml) for 30 min at 37 °C. Cells were activated with WKYMVm (100 nM, A) or galectin-3 (40 µg/ml, B) and the release of superoxide was recorded continuously. The data is expressed as fold increase of superoxide release from TNFα primed neutrophils compared to control cells received no TNFα (peak values from TNFα primed and non-primed were used for fold increase calculation). Each symbol represents one individual mouse. One-way ANOVA followed by Turkey׳s multiple comparisons test, n.s, not significant. The dashed lines indicate no increase (fold increase 1).

Data describes mouse neutrophil NADPH-oxidase activation measured by isoluminol-enhanced chemiluminescence systems, upon stimulation with the formyl peptide receptor (Fpr) agonist WKYMVm (100 nM) or galectin-3 (40 µg/ml) [1], [2], [3], [4]. Neutrophils were isolated from four different mouse strains C57BL/6, DBA/1, BALB/c and NMRI. Real time kinetics of superoxide release induced by WKYMVm and galectin-3 from strain C57BL/6 are shown (Figs. 1 and 2). The maximal levels of superoxide release from neutrophils with four different strains are shown (Figs. 1 and 2). In addition, the NADPH-oxidase activity in bone marrow neutrophils primed with TNFα acting through the TNFRI [5] is presented in Fig. 3.

## 2 Experimental design, materials and methods

### 2.1 Mice

Female C57BL/6, DBA/1, BALB/c and NMRI mice between 10–20 weeks of age were used in this study. Mice were purchased from B&K Universal AB (Stockholm, Sweden) and maintained under pathogen-free conditions in the animal facility of the Department of Rheumatology and Inflammation Research, Gothenburg University. The animal study was approved by the Ethical Committee for Animal Experimentation, Gothenburg, Sweden.

### 2.2 Chemicals

WKYMVm was from AltaBioscience (University of Birmingham, Birmingham, UK). Horse radish peroxidase (HRP) was from Boehringer Mannheim (Mannheim, Germany). Isoluminol and uric acid were from Sigma Chemical Co. (St. Louis, MO). Recombinant mouse TNFα was from R&D Systems (Abingdon, Oxon, UK) and diluted in PBS containing 1% BSA. All subsequent dilutions of reagents were made in Krebs-Ringer phosphate buffer that was supplemented with glucose (10 mM), Ca^2+^ (1 mM), and Mg^2+^ (1.5 mM) (KRG; pH 7.3) prior to use. Recombinant galectin-3 was produced in *Escherichia coli* and collected by affinity purification on a lactosylsepharose column and stored at 4 °C in phosphate-buffered saline (PBS, pH 7.2) containing 150 mM lactose until further purification by gel filtration (PD10, Pharmacia, Uppsala, Sweden) to remove lactose. Endotoxin was reduced to <10 pg/μg of galectin-3 (as determined by Limulus aembocyte lysate assay) using an AffinityPak^™^ Detoxin-gel^™^ column (Pierce, Rockford, USA).

### 2.3 Mouse neutrophil separation

Mouse neutrophils were isolated from bone marrow as described earlier [6]. Briefly, femur and tibias were removed and flushed through with ice cold KRG without Ca^2+^, to obtain bone marrow cell suspension. The mix were suspended to a single cell solution, filtered and pelleted by centrifugation on a three layer percoll gradient 1.095, 1.085 and 1.070 g/ml. This isolation procedure routinely gives 95% purity of neutrophils as determined by May-Grünwald and Giemsa staining. Peritoneal exudated neutrophils were obtained from peritoneal cavity 4 h post uric acid (10% in NaCl, 100 *µ*l) injection.

### 2.4 The NADPH-oxidase assay

The extracellular release of ROS was recorded using a six-channel Biolumat LB9505 apparatus (Bertold Co., Wildbad, Germany) and an isoluminol amplified chemiluminescence (CL) technique as described earlier [7]. The reaction mixture of 0.9 ml containing 5×10^4^ cells, horse radish peroxidase (HRP; 4 U) and isoluminol (20 µM) was equilibrated in the luminometer for 5 min at 37 °C, after which the stimulus (0.l ml) was added. The extracellular production of superoxide anions from activated neutrophils was recorded continuously by light emission (counts per minute). *in vitro* priming was achieved by incubation with the priming agent TNFα (50 ng/ml) at 37 °C 30 min prior to the addition of a stimulus [5].

### 2.5 Statistical analysis

One-way ANOVA followed by Turkey׳s multiple comparisons test was used for statistical analysis. A *p* value ≤0.05 was considered statistical significant. n.s: not significant.
